# Application of Broccoli Stalk Powder in Bread Formulations

**DOI:** 10.3390/molecules31091414

**Published:** 2026-04-24

**Authors:** Elena Roxana Margarit, Andreea Antonia Georgescu, Elena Corina Popescu, Aslıhan Tüğen, Claudia Lavinia Buruleanu

**Affiliations:** 1 Faculty of Environmental Engineering and Food Science, Valahia University of Targoviste, 13 Sinaia Alley, 130004 Targoviste, Romania; elena.margarit@valahia.ro (E.R.M.); andreea.georgescu@valahia.ro (A.A.G.); corina.popescu@valahia.ro (E.C.P.); 2 Department of Food Engineering, Institute of Sciences, Afyon Kocatepe University, 03200 Afyonkarahisar, Türkiye; aslihan.tugen@usr.aku.edu.tr

**Keywords:** broccoli stalk powder, antioxidants, bread quality, sensory acceptance

## Abstract

Bread enriched with broccoli stalk powder is proposed as a newly formulated product with potential health benefits. Wheat flour in the bread recipe was enriched with powder obtained from freeze-drying broccoli stalks, a valuable by-product of vegetable processing. The effects of broccoli stalk powder (BSP) supplementation on the physicochemical and sensory properties of bread, as well as its bioactive profile, were evaluated. The results showed an increase in moisture content and acidity with increasing substitution levels from 0% (control bread—BC) to 7%, while some important parameters in terms of consumers’ acceptability decreased (i.e., loaf volume and porosity). Elasticity exhibited moderate variations, with no major influence at lower substitution levels. A small-scale consumer test indicated good scores up to moderate substitution levels (3–5%). The antioxidant activity of broccoli stalk flour (62.13% ± 1.29%) positively influenced the antioxidant activity of bread with 3% BSP, which increased by approximately 4%. The total polyphenol content (TPC) of the bread with 5% BSP, together with its physicochemical and sensory characteristics, suggested that broccoli stalk powder is a promising functional ingredient for bakery applications.

## 1. Introduction

Global food systems are confronting major sustainability challenges arising from population growth, climate change, and resource scarcity. Sustainable food systems are defined as systems that balance environmental, economic, and social dimensions, meeting present needs without jeopardizing the capacity of future generations to meet their own needs [1]. Within this framework, the circular economy concept is increasingly displacing conventional linear “take–make–dispose” models. This model centers on closed-loop mechanisms that aim to retain resources within the system at the highest efficiency and for the longest possible duration, minimize waste generation, and reprocess by-products to reintegrate them into the economy [2]. The emergence of substantial quantities of by-products and waste during food production and processing positions the circular economy approach as critical for the food industry.

Moreover, the increasing consumer demand for safer and health-promoting foods encourages the use of naturally derived by-product components as alternatives to synthetic additives [3]. This trend highlights the strategic importance of vegetable by-products in addressing food insecurity and enhancing nutritional security.

The bakery sector is among the largest global food sectors and consumes considerable volumes of raw materials. Traditionally, the sector has relied predominantly on staple inputs such as wheat flour, fat, and water. However, increasing consumer awareness and the demand for healthier diets are steering the sector towards the development of more nutritious and functional products. In recent years, the enrichment of bread with functional compounds that impart health-promoting and preventive effects has attracted significant global attention [4]. Concurrently, the need to improve the sector’s sustainability performance has necessitated the exploration of alternative and innovative raw material sources [5].

The bakery sector constitutes one of the most widely consumed food categories worldwide and therefore offers an ideal platform for delivering functional constituents to consumers. The low fiber content, high glycemic index, and limited bioactive compound profile of conventional baked goods create a clear imperative to enhance their nutritional quality. The incorporation of vegetable by-products into bakery products provides a dual benefit by improving nutritional quality while reducing food waste [1,4,5,6].

Transforming by-products into functional foods through appropriate processing techniques and hygiene protocols is critical for preserving the physical and organoleptic properties of the products. Nevertheless, during incorporation into food formulations, potential changes in color, texture, and overall consumer acceptability must be optimized [7]. In this context, technological approaches developed to address these issues facilitate the industrial-scale adoption of by-products and help overcome barriers associated with high operational costs.

The incorporation of vegetable processing by-products into bread as functional ingredients has been extensively investigated, not only from a sustainability perspective but also due to their potential to increase the nutritional density and bioactive richness of the final product. Within defined substitution levels, statistically and technologically meaningful increases can be achieved in the phenolic compounds, antioxidant capacity, dietary fiber, and certain minerals. However, as the substitution ratio increases, technology- and quality-related trade-offs become more pronounced, including color darkening, reduced product volume, increased textural firmness, and a concomitant decline in sensory acceptance [7,8]. Consequently, the practical challenge is to define, for each specific by-product, an optimal balance between the substitution range that maximizes bioactive gain and the sensory/technological quality level that remains acceptable.

Several studies have been conducted to develop functional bread. Enhancing the nutritional value of bread through enrichment with microalgae (spirulina and chlorella) [4], marine shellfishery products [9], broccoli sprouts [10], broccoli leaf powder [11], sea buckthorn berry [12], chickpea flour [13], kale [14], and lemon pomace fiber [15] has been widely reported.

Cruciferous vegetables including broccoli are known for their bioactive compounds with potent antibacterial and antioxidant properties. Bioactive substances from broccoli and broccoli by-products have been shown to exert cancer-preventive effects [16,17], lower glucose levels [18], and reduce inflammation and oxidative stress, which are key factors in cardiovascular disease [19]. Functional products enriched with dietary fiber and antioxidants contribute significantly to consumer health [20]. However, further animal studies are needed to validate the therapeutic potential of broccoli-derived bioactive compounds [16].

Broccoli (*Brassica oleracea* var. *italica*) is an agricultural crop belonging to the *Brassicaceae* family and originating from the Mediterranean region, with significant social and economic importance. It is now cultivated worldwide across a wide range of climates and regions, both in open fields and in greenhouse production systems [21], resulting in a considerable diversity among broccoli varieties [16]. In 2023, global broccoli production reached 26,472,040 thousand tons, representing a 1.65% increase from 2019 [22].

Marketable broccoli florets account for only a small fraction of the total above-ground plant biomass [23]. Thus, broccoli production generates considerable waste materials, such as leaves, stalks, and non-marketable vegetables [24,25]. Currently, more than 60% of global broccoli production is wasted during harvesting [26], resulting in environmental pollution [16,27,28]. The valorization of broccoli waste aligns with the Sustainable Development Goals (SDGs) initiative, contributing to SDG 12 (Responsible Consumption and Production) by reducing food loss, SDG 13 (Climate Action) by mitigating waste-related emissions, and SDG 2 (Zero Hunger) by enhancing global food security [29].

Research on broccoli has predominantly focused on its edible parts, whereas investigations into its by-products remain scarce [26]. However, various anatomical components of the broccoli plant have been documented as being incorporated into the formulation of novel functional food. Several foods, such as gluten-free cake [20], biscuits [25], gluten-free mini sponge cakes [30], crackers [31], pasta [32,33], noodles [34], spreadable cheese [35], salad dressing [36], and béchamel sauce [37] have been successfully formulated using broccoli co-products in recipes.

The valorization of broccoli by-product extracts has emerged as a promising approach, primarily attributed to the high dietary fiber (DF) content of the stalks [38]. The chemical composition of the broccoli stalk includes both soluble and insoluble fibers (pectins, cellulose, starch, hemicellulose, etc.), vitamins (including some B vitamins and ascorbic acid), minerals (K, Ca, Zn, Mg, P, Fe, Mn, etc.), flavonoids, and amino acids (including cysteine, a sulfur-containing amino acid) [23]. Compared to the floret tissue, the stem tissue contains a significantly lower total glucosinolate concentration. Nevertheless, the percentage of glucoraphanin to total glucosinolates in the stem indicates that it contains considerable amounts of health-promoting compounds [39]. Also, sulforaphane, a bioactive compound resulting from the hydrolysis of glucoraphanin, was reported in high amounts in the stem of some broccoli cultivars [40].

Genetic, geographical, and environmental factors may account for significant variations in the nutritional composition of the different botanical parts of broccoli, including the stems. The proximate composition of broccoli stalks was reported as follows: moisture 85 g/100 g FW, ash 10.34–12.37 g/100 g DW, protein 13.73–18.14 g/100 g DW, carbohydrate 75.42 g/100 g DW, total dietary fiber 77.28 g/100 g DW (8.30 g/100 g FW), and lipids 6.58 g/100 g DW [38]. To encourage the use of broccoli stalk, it is important to assess its nutritional value for human consumption [39] and also the mechanisms of action of its bioactive substances [16].

The conversion of broccoli by-products into functional ingredients for food applications exemplifies a circular economy strategy and provides a cost-effective means of valorization [25]. The transition from waste to a valuable resource faces challenges such as the scalability and cost-effectiveness of processing technologies and seasonal availability of broccoli by-products [29]. These challenges underscore the need for further research to fully realize the potential of broccoli by-product valorization.

Accordingly, this paper aims to examine the potential use of freeze-dried broccoli stalk in the bakery to produce functional bread with enhanced phenolic compounds and dietary fiber and to study the influence of its inclusion on the physico-chemical parameters and sensory attributes of bread, which are critical for consumers’ acceptance.

## 2. Results

### 2.1. Physico-Chemical Parameters of Flours

The moisture contents of the broccoli stalk powder and wheat flour used in experimental were 6.23 ± 0.01% and 13.56 ± 0.05%, respectively. The hydration capacity, wet gluten, deformation index (DI), and elasticity index (I_e_) were determined (Table 1).

### 2.2. Dough Properties During Mixing and Fermentation

Farinographic analysis provided the data needed for designing the enriched breads, namely water absorption of the wheat flour, defined as the amount of water required to achieve standard dough consistency, the development time, the duration of peak consistency, and dough softening after mixing. These key parameters are indicative of the gluten strength and mixing tolerance.

The correlation of farinographic and alveographic parameters (Table 2) consistently highlights the profile of a flour of medium rheological intensity, characterized by moderate gluten in terms of both strength and stability.

The Falling number of the wheat flour used in the experiment was 269 s. Although the value is at the lower limit of the optimal range for baking (250–350 s), it results that the alpha amylase activity in the flour had an optimum activity to support breadmaking, in accordance with the results provided by the farinogram and alveogram.

### 2.3. Physico-Chemical Parameters of Bread

**Figure 1:**
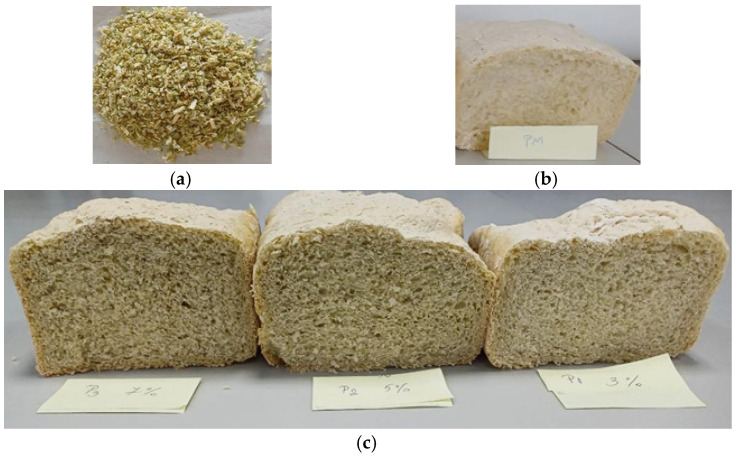
Aspect of the freeze-dried broccoli stalk powder (**a**) and experimental bread: control (**b**) and bread obtained through the enrichment of wheat flour with broccoli stalk powder (**c**). In figure (**c**), the supplementation of wheat flour with broccoli stalk powder decreased from left to right.

Figure 1 shows images of cross-sections of experimental bread obtained with and without broccoli stalk powder (BSP).

The moisture content of the bread increased with higher levels of broccoli powder added to wheat flour (Table 3), ranging from 5.72% (B3BSP) to 12.59% (B7BSP), when the control sample (BC) was included. The dietary fibers, non-starch polysaccharides, and other structural compounds present in broccoli stalk flour exhibit high water-binding capacity, which likely accounts for the observed increase in bread moisture. Enhanced water retention within the dough matrix contributes to a more hydrated crumb structure. Furthermore, the reduction in the gluten proportion resulting from the enrichment of wheat flour with BSP may alter the protein network of the dough, thereby facilitating additional water retention.

One-way ANOVA revealed a statistically significant effect of BSP on the moisture content of bread (*p* < 0.001). Post hoc analysis (Tukey’-b test) indicated significant differences (*p* < 0.05) between BC and bread with enrichment levels of BSP (3%, 5%, and 7%, respectively), confirming that BSP significantly increased water retention capacity in bread formulations. From a technological perspective, moisture is highly sensitive to recipe changes involving fiber-rich ingredients.

As well as the moisture, the bread acidity increased with higher addition levels of broccoli stalk powder (Table 3), with this trend being manifested at 5% and 7% supplementation. Thus, the acidity of the B5BSP sample was 1.14 times higher than the control, while that of the B7BSP sample was 1.23 higher compared to the same referential. Tukey analysis indicated significant differences (*p* < 0.01) between BC and B5BSP and between BC and B7BSP. This increase may be associated with the biochemical composition of BSP, mainly the presence of organic acids (i.e., malic acid, citric acid), and its influence on the fermentation dynamics of the dough.

The porosity of the experimental bread decreased compared to the control sample (Table 3), affecting the crumb structure. This reduction is dramatic, by an average of 20% reported for the control, and starts from the lower level of BSP supplementation, probably due to the addition of non-gluten material and its negative influence on the gluten network development. According to ANOVA analysis, decreasing the bread porosity with increasing BSP addition levels is statistically significant (*p* < 0.001).

The loaf volume of bread decreased with increasing BSP levels (Table 3), from 320.66 ± 1.15 cm^3^ (BC) to 238.66 ± 1.52 cm^3^ (B7BSP). ANOVA analysis showed a significant decrease in bread volume with an increasing substitution level (*p* < 0.001). This parameter is strongly related to gluten network development and bread porosity, as wheat gluten proteins are responsible for forming the viscoelastic network able to retain the gases resulting from fermentation.

The loaf volume was identified as a sensitive technological parameter, affected by wheat flour addition, whereas bread elasticity remained comparatively stable, showing only moderate variations among samples (Table 3). Elasticity values suggest that the levels of BSP used did not significantly compromise the crumb resilience. It can be assumed that some interactions between dietary fibers and water counterbalanced for gluten reduction. ANOVA analysis showed no statistically significant effect (*p* > 0.05) of BSP supplementation on bread elasticity. These findings suggest that elasticity is less sensitive to broccoli stalk flour substitution than the bread volume and porosity.

### 2.4. Effect of Broccoli Stalk Powder Addition on the Fiber Content

**Figure 2:**
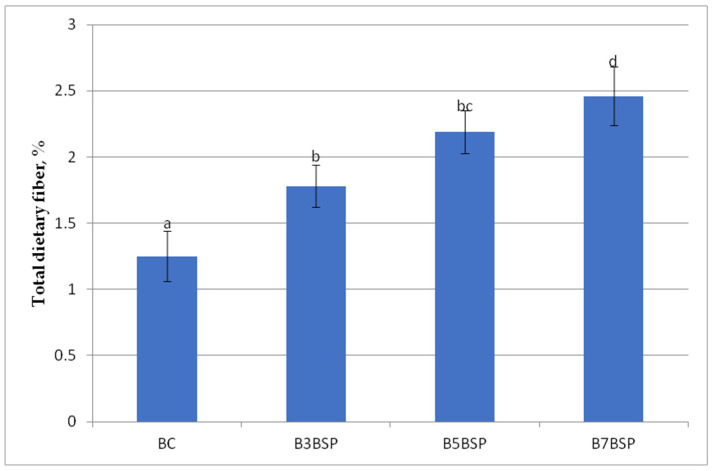
Total dietary fiber of experimental bread. Different lowercase letters within each sample represent significant differences according to Tukey’s-b test (*p* < 0.05).

The dietary fiber content of the experimental bread increased with the addition of BSP (Figure 2), due to the high content of insoluble fiber (cellulose, hemicellulose) and soluble fiber (pectin) in broccoli stalks. From a technological perspective, increasing BSP addition enhanced the water-retention capacity, as previously described.

Even a small addition (only 3% BSP) caused a visible increase in TDF, by 42% compared to the control. The supplementation of wheat flour with BSP at a 7% level resulted in a fiber content nearly double that of the control. The increase in the fiber content of bread with the addition of BSP is not linear, but it is consistent and significant. ANOVA analysis revealed significant differences between the means due to the addition of BSP to the wheat flour. The control (BC) differed significantly from all other samples (*p* < 0.05), whereas the addition of 3% and 5% BSP to the wheat flour did not result in significant changes in the fiber content of the breads. A post-hoc test indicated that sample B7BSP differed significantly (*p* < 0.05) from the other samples. The results highlight the potential of BSP as a functional ingredient for improving the nutritional value of bakery products.

The addition of broccoli fiber to bread may provide additional benefits compared to cereal fiber, such as potential prebiotic effect through the stimulation of beneficial microbiota, reduction of the glycemic index of bread, and increased satiety due to the water-retention capacity.

### 2.5. Effect of Broccoli Stalk Powder on Total Polyphenol Content (TPC) and Antioxidant Activity (AA) of Bread

**Figure 3:**
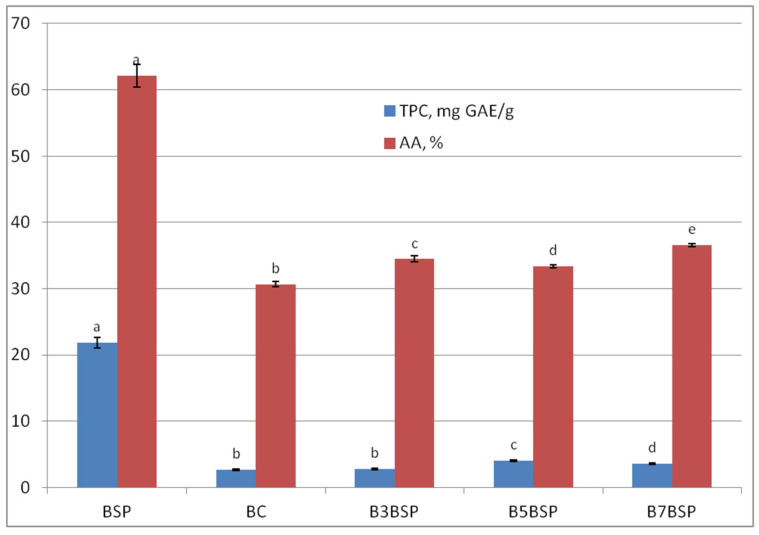
Antioxidant activity and total polyphenol content of broccoli stalk powder and bread samples. Different lowercase letters within each parameter (TPC and AA respectively) and for each sample represent significant differences according to Tukey’s-b test (*p* < 0.05).

Broccoli stalk powder was characterized by high antioxidant activity (62.13 ± 2.53%) and total polyphenol content (21.8 ± 1.19 mg GAE/g), with freeze drying used to protect the bioactive compounds. The incorporation of broccoli stalk powder significantly influenced both the antioxidant activity (AA) and total polyphenol content (TPC) of bread samples (Figure 3), consistent with the findings reported by Lafarga et al. [41].

Increasing the substitution level of BSP led to increased values of AA and TPC, although this trend was not progressive for any of the parameters. Thus, the AA of bread with 7% BSP increased by approximately 20% compared with the control, while the TPC of bread with 5% BSP increased by 51.7% compared with the control. Positive correlations between AA and TPC have been reported, although the correlation coefficients varied relatively largely depending on the method used to determine AA, such as DPPH and FRAP [31]. No significant differences (*p* < 0.05) were determined between the total polyphenol content of BC and B3BSP. In terms of antioxidant activity, all samples were significantly different (*p* < 0.05).

The TPC content of the experimental sample increased at 3% and 5% supplementation with BSP, respectively, while a decreasing trend was observed at a high level (7%) of supplementation. This can be due to a different degree of inactivation of polyphenol oxidase at elevated temperatures [42].

### 2.6. Small-Scale Consumer Test and Colorimetric Analysis of Bread

**Figure 4:**
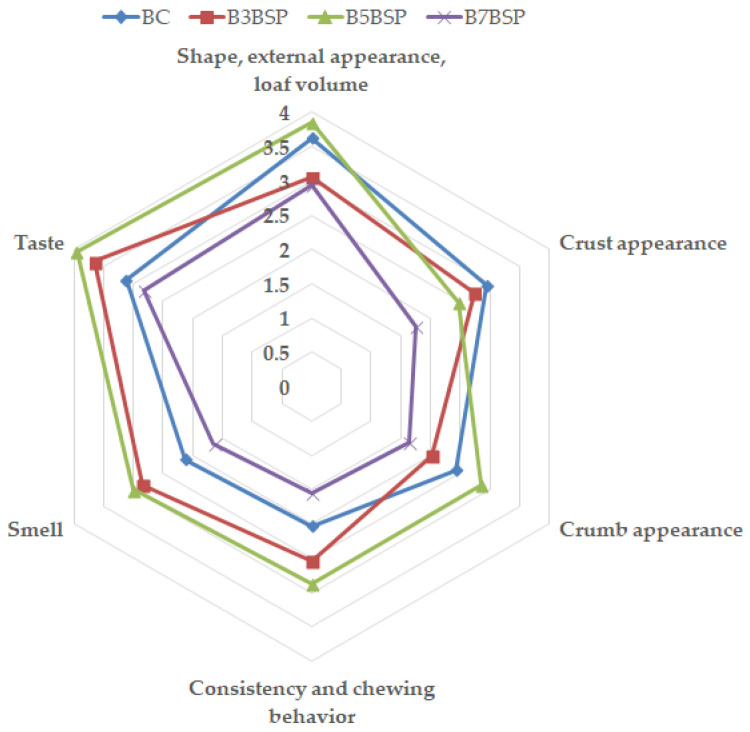
Sensory evaluation of control and BSP-enriched breads.

The Small-scale Consumer Test was conducted in accordance with the Informed Consent and Ethics Statement for research involving human participants, as described below. The test indicated good acceptability of breads for 3% and 5% supplementation levels (Figure 4), in agreement with previous studies on the inclusion of broccoli powder in bread [10]. In contrast, a higher level of BSP (7%) negatively affected all visual and textural properties. The pronounced greenish color of this sample and its increased crumb density had low scores.

Lightness is an important characteristic in bread, particularly for assessing product quality and consumer acceptability. It reflects flour quality and influences consumer perception, as visual appearance is one of the primary criteria in choosing the assortment of bread. The higher lightness observed in fortified bread compared with the control may be attributed to the pale green color of BSF, which causes the dilution of the carotenoids in flour. Additionally, the fiber content of broccoli stalk flour may affect how the bread surface reflects light.

The L* values (Table 4) suggest a lighter crumb color in all samples with incorporated broccoli powder compared to the control, probably due to a reduced extent of Maillard reactions [41].

All L* values were higher than 50, indicating a relative light crumb color. Changes in crumb color characteristics were attributed to supplementation with BSP, although no direct relationship between the level of addition and L* values was observed, as was also the case for a* and b* values. The observed differences in L* values are likely associated with variations in the composition [43].

Negative a* values were determined for all fortified breads, indicating a green color. The positive b* values increased with increasing levels of addition, indicating a shift toward yellow coloration.

Delta E (ΔE) is a quantitative metric that expresses the difference between two colors, indicating how distinguishable they are to the human eye. In brief, ΔE represents the “distance” between two colors within a color space, typically the CIELAB system. The ΔE values for breads formulated with broccoli stalk powder were 10.89 for the 3% BSP formulation, 9.27 for the 5% BSP formulation, and 11.61 for the 7% BSP formulation.

### 2.7. Correlation Analysis of Chemical Parameters and Colorimetric Attributes

Pearson correlation analysis (Table 5) was applied to provide an integrated view on the chemical parameters characterizing the experimental bread.

Pearson correlation analysis revealed strong negative correlations between bread moisture and volume, bread volume and AA, and bread volume and TDF (*p* < 0.01). Strong positive correlations were determined between moisture and acidity, moisture and AA, and moisture and TDF (*p* < 0.01). A significant moderate positive correlation between TPC and moisture (r = 0.795, *p* < 0.01) was also established. No correlation was found between AA and TPC, possibly due to the influence of other chemical constituents on the antioxidant activity of bread. According to Zhao et al. [28], flavonoids are the primary contributors to the antioxidant activity in broccoli floret and broccoli by-products, including the stalk. In contrast, a strong correlation has been observed between TPC and DPPH-radical-scavenging activity in broccoli by-products [40].

Strong positive correlations were found between TDF and antioxidant activity (r = 0.834, *p* < 0.01), as well as TDF and TPC (r = 0.782, *p* < 0.01). These associations underline that broccoli stalk powder contributed both to the fiber enrichment of breads and to an increased content of bioactive compounds. The results are consistent with those reported by Quizhpe et al. [44].

**Figure 5:**
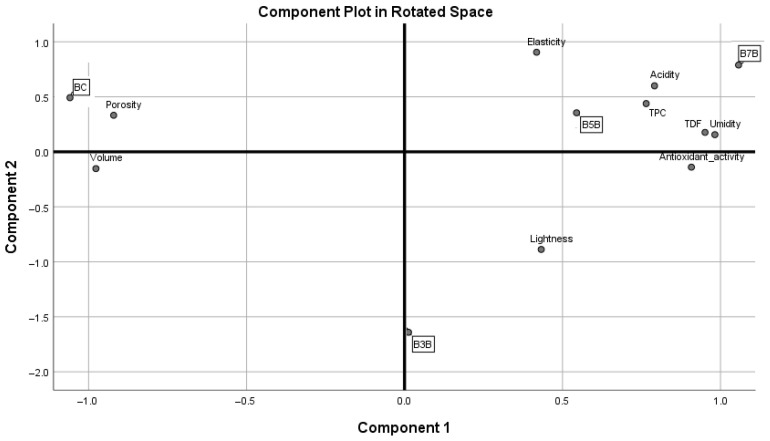
Principal Component Analysis.

Principal Component Analysis (Figure 5, Table 6) was applied to correlate the results of the physicochemical analysis with the profile of the four experimental breads. Principal component 1 (PC1) accounts for 67.425% of the variance, while Principal component 2 (PC2) accounted for 26.294% of the variance.

Moisture, acidity, antioxidant activity, total polyphenol content, and total dietary fiber are positioned in the positive part of PC1. This component is negatively associated with porosity and bread volume, indicating that PC1 characterizes breads with less porous and less voluminous structures.

Bread elasticity, as previously described in relation to the influence of BSP supplementation, had a high loading in the positive part of PC2. Component 2 was also primarily defined by lightness (negative loading), indicating a contrast between rheological and visual properties. Samples with higher elasticity tended to exhibit lower lightness values.

Overall, the PCA indicates that the main sources of variation arise from differences in compositional and structural characteristics (PC1) and from texture–appearance relationships (PC 2), highlighting the complexity and multidimensional nature of the breads.

Although bread porosity is important for consumer acceptance, this vector is located close to the control (BC). Figure 5 highlights the positive influence of wheat flour supplementation with 5% and 7% BSP respectively on the main physico-chemical and bioactive characteristics of the product. PC1 shows that composition and structure are the main drivers of variation, while PC2 indicates differences in texture and appearance. The control sample (BC) was structurally light/porous but firm, with lower moisture, TDF content, and antioxidant activity. The sample enriched with 3% BSP was located at the bottom center, characterized by a moderate chemical composition, low elasticity, and lighter color, suggesting that further optimization of the composition and/or texture is needed. In contrast, the samples enriched with 3% BSP and 5% BSP were positioned in the top-right region, exhibiting high elasticity and high values of the chemical parameters (moisture, TDF, and antioxidant activity). Thus, B5BSP and B7BSP combine high nutritional content with good texture.

## 3. Discussion

The flour used in the experiments presented a balanced rheological profile, with medium-strength gluten, capable of supporting standard technological processes. The farinogram–alveogram correlation confirmed that the analyzed flour is suitable for current bakery products, with relatively short processes and moderate hydration, where the P/L balance and rapid dough development are exploited. Accordingly, because the wheat flour had medium-strength gluten, it was assumed that the dough structure can absorb the addition of broccoli stalk powder without compromising its cohesion and extensibility. In moderate doses (e.g., 5–10%), broccoli fiber can improve the water-holding capacity, contributing to moister breads with improved softness. Dough supplemented with BSP required slightly longer and more careful kneading to properly activate the gluten [45]. Based on previous findings, the supplementation of wheat flour with BSP did not exceed 10%.

Previous studies have reported an increase in the moisture content of bread following the incorporation of powdered tomato co-products into their formulations [46]. Sayed-Ahmad et al. [47] observed an increase in the moisture content of bread following its fortification with chia seed flour, which was attributed to the higher content of water-soluble fiber in the chia-enriched bread.

The starch degradation, closely related to the water content of bread, is known as being responsible for the bread’s staleness. Fiber incorporation into the dough through BSP supplementation enhances hydration of the crumb matrix, potentially contributing to the bread texture, staling retard, and improved freshness retention. However, the shelf life of the product could be threatened because high levels of moisture can negatively influence the microbiological stability, so that optimization of technological parameters, packaging, and storage processes will play a decisive role in both scopes.

High addition levels of BSP lead to modification of the carbohydrate composition of dough and consequently to changes in fermentation activity of the yeast. Thus, BSP brings fiber that can slow down fermentation, resulting in lower acidity in some cases. By another hand, the nutritional compounds can stimulate microbiota, with the effect being the high production of organic acids. Not in the last, the proteins from broccoli stalk flour can increase the buffering capacity of the dough. Supplementing wheat flour with BSP tends to increase the bread acidity, but the effect depends on the level of addition and its impact on yeast and lactic acid bacteria responsible for alcoholic fermentation and lactic acid fermentation of the dough respectively.

In terms of consumers’ acceptability and shelf life of the product, high acidity levels may affect both the flavor profile of bread and its microbial stability. Although the effect of acidity appeared to be less pronounced than that on the loaf volume and porosity of bread, it remains technologically relevant. Finally, the moisture and acidity of bread behave increased proportionally with the level of fiber inclusion from broccoli stalk, suggesting that both hydration and biochemical effects are dose-dependent.

The gluten dilution due to the enrichment of wheat flour with broccoli stalk powder led to weakened gas-retention capacity of the dough [48]. Reducing the gas retention as a result of the disruption of the gluten network by BSP also led to a denser crumb structure of the bread, in opposition to its porosity. Korus et al. [14] observed a decrease in porosity of the bread enriched with powdered kale, but compared with the control, the differences were non-significant.

Interactions between phenolic compounds and gluten proteins have been reported to be responsible for various effects on the microstructure of bread and several of its sensory properties [49]. High concentrations of tea polyphenols decrease disulfide bonds, weaken the gluten network, and also reduce the loaf volume [50]. Similarly, a decrease in the loaf volume of vegetable-flour-loaded bread was determined by Mastromatteo et al. [51], Wang et al. [52], and González-Montemayor et al. [53]. The texture, an essential sensory attribute, was significantly influenced by the addition of celery powder, causing an increase in firmness and a denser core structure [52].

In agreement with the findings of this study, adding powdered broccoli co-products at a 2% (*w*/*w*) level to bread formulations resulted in a reduction in the specific volume compared to the control sample [41]. Similar results were reported by Lee [54], Anwar et al. [55], and Lafarga et al. [9]. This effect has been attributed to the dilution of starch and gluten following the addition of broccoli powder, as well as by a reduction in fully hydrated starch granules due to competition for water between the powder and the starch [41]. Furthermore, broccoli co-products are rich in glucosinolates, compounds with strong antimicrobial activity; their incorporation into bread formulations may therefore modulate yeast performance, potentially affecting the bread volume even at low inclusion levels [41]. 

Whole legume flours (green beans, peas, mesquite), rich in dietary fiber, contributed to increasing the fiber content of sourdough bread but moderately to the antioxidant activity of the bread. The additions produced a denser bread texture and higher water absorption of the dough [53]. Also, fibers isolated from broccoli stalks demonstrated a high water-absorption capacity, increasing the firmness of the bread crumb and reducing the elasticity of the dough. They contribute to a denser crumb structure. Fibers can dilute the gluten network, likely resulting in a decrease in specific volume and a more compact crumb structure. Fiber interaction with gluten may require a hydration adjustment and formulation optimization. Not in the end, dietary-fiber-rich fractions isolated from broccoli stalks may induce darker/greener hues [56].

The composition of broccoli dietary fiber (DF) is complex. The insoluble dietary fiber (IDF) in broccoli is predominantly composed of cellulose, hemicellulose, and lignin, whereas its soluble dietary fiber (SDF) comprises pectins, gums, mucilages, and β-glucans. The contents of IDF and SDF are also influenced by the broccoli cultivar [45]. However, the functional properties of individual components of DF remain insufficiently elucidated [27]. Consistent with total dietary fiber content, broccoli stems contain higher levels of both insoluble dietary fiber (IDF) and soluble dietary fiber (SDF) compared to leaves and inflorescences, with IDF representing the predominant fraction due to the physiological functions of the stalks [38,45]. Unlike SDF, IDF does not form gels upon contact with water, but is capable of retaining water within its structural matrix, thereby enhancing intestinal transit [38].

The total dietary fiber (TDF) of broccoli stalk powder was high, by 33.58 ± 1.31%, making it an excellent raw material for product enrichment. The processing method did not result in a significant change in the total fiber content of broccoli stems subjected to hot air drying; nevertheless, lower fiber content was observed in samples derived from lyophilized stems [45]. The cruciferous-based residues, including those derived from broccoli, can be efficiently valorized through innovative extraction and biotransformation techniques [57]. Extraction methods need to be optimized to obtain functional fibers. Accordingly, green extraction methods have been applied to obtain novel fiber-rich ingredients, with antioxidant and technological properties from broccoli stalks [56].

The incorporation of vegetable flours, including carrot, tomato, beetroot, and broccoli, into bread has been shown to enhance antioxidant levels [48]. Studies have revealed that extracts derived from broccoli stems possess higher antioxidant activity than those from florets [29]. Furthermore, although beneficial compounds present in cruciferous vegetables may be significantly reduced during thermal processing, some studies indicate that heat-sensitive ingredients can exhibit greater stability when incorporated into baked products [58]. The antioxidant properties of broccoli by-products are related to their comprehensive phytochemical profile. For example, they were found to be abundant in sulforaphane, an isothiocyanate renowned for its potent antioxidant properties [45], as the high content of value-added compounds, such as vitamins and phenolic compounds, gives broccoli and its various parts beneficial properties, including antioxidant activity [38]. It has been also suggested that the insoluble dietary fiber, which is present in high amounts in broccoli stalks, possesses the ability to associate with phenolic compounds through both covalent and non-covalent interactions, thereby serving as a structural matrix for the retention of these bioactive molecules [59].

The total phenolic concentration in stems, by 1.41 mg GAE/g DW, was 2.9-fold lower than TPC found in broccoli leaves [39]. The authors determined that broccoli stem tissue exhibited a DPPH-radical-scavenging activity of 16.4%. In another study, the TPC of broccoli stems was reported as 312 ± 41 mg/100 g DW [41], with a moisture content of 90.1 ± 1.4% for the stems used in breadmaking

Large variation among cultivars in terms of phenolic compounds and ROS-scavenging capacity has been reported [40]. Therefore, substantial genetic variation in broccoli tissue antioxidants should be considered by growers and breeders to enhance sustainability and productivity, particularly for by-product utilization [39]. Contrary to the abovementioned data reported by Liu et al. [39], higher TPC was found in broccoli stalks, reaching 9.39 mg GAE/g DW [38].

It has been observed that adding broccoli to bread affects oxidative stability during gastrointestinal digestion [60], while broccoli leaf powder significantly improves the antioxidant potential of gluten-free bread [11]. The incorporation of freeze-dried broccoli co-products into crackers has been reported to significantly increase the TPC and antioxidant capacity [14]. Gawlik-Dziki et al. [10] stated that incorporating broccoli sprouts into wheat bread at levels of 1–5% (*w*/*w*) led to an increase in antioxidant capacity and the significant enrichment with phenolic compounds; however the relationship between TPC and the percentage of addition was far from linear [10]. Similarly, Lee et al. [54] determined DPPH·-scavenging activity ranging from17.6% to 45.5% when broccoli was incorporated into breads at 2.5–10.0% (*w*/*w*).

The increases observed in antioxidant capacity and TPC in some experimental samples can be attributed to enzymatic action on the plant matrix [44]. This process degrades cell wall components, releasing bound polyphenols, which are subsequently converted into soluble and more bioavailable forms. However, although phenolic compounds are generally resistant to heat, their levels in the final product may decrease due to the breakdown of phenolics during processing [33]. Furthermore, the retention of bioactive compounds is strongly influenced by processing conditions, which may explain why the enriched breads did not exhibit significantly higher antioxidant activity or phenolic content compared to the control [61].

Incorporating vegetables into bread is generally well received by consumers and represents a convenient strategy to increase vegetable consumption [62] while delivering plant-based ingredients with demonstrated health effects [48]. Texture-related sensory changes in bread are primarily caused by interactions between phenolic compounds or other antioxidants present in extracts from flours obtained from agri-food industry by-products and gluten proteins. These antioxidants can reduce disulfide bonds, which play a crucial role in maintaining the structure of the gluten network [49].

In agreement with our results, Anwar et al. [55] reported that breads containing 1–2% (*w*/*w*) broccoli achieved the highest overall acceptability scores, while higher concentrations resulted in unacceptable textural and sensory properties. Additionally, bread formulations containing 2% (*w*/*w*) powdered broccoli stalks maintained their acceptability, appearance, and texture [41]. The overall sensory acceptability of gluten-free mini sponge cakes also decreased with increasing levels of broccoli leaf powder [30].

The color changes were consistent with findings reported in the literature, which show that incorporating vegetables or by-products from the vegetable industry into bakery formulations significantly affects product color [46]. Accordingly, the incorporation of powdered broccoli stalks [10] and broccoli sprouts [41] also resulted in lower a* values in bread compared to the control. Similarly, such breads exhibited significantly more negative a* values and higher b* values than the control bread, indicating a greater contribution of green and yellow hues [11]. It was reported that breads containing broccoli exhibited a more pronounced green coloration, but the incorporation of broccoli did not affect the overall acceptability of the breads [41].

Color is a key quality parameter that significantly influences the consumer acceptance of food products [20,43]. Broccoli flour retains the carotenoids and chlorophylls of the vegetable, which confer an attractive green color when incorporated into food formulations [25]. However, β-carotene, which is most abundant in florets and leaves, is not present in stalks [38]. If broccoli is heat-processed before use, its behavior in dough may change, indirectly influencing the texture, crumb stability, and final color of the bread [42].

During the baking process, a range of compounds is formed as a result of chemical reactions, such as caramelization and Maillard reactions, leading to changes in the color of the final product. Consequently, variations in protein content and phenolic compounds may influence the lightness of the finished products [20,43]. A decrease in the L* value of breads containing broccoli powder compared to the control was obtained by Lee [54]. However, the broccoli powder improved the overall nutritional value of the bread.

Finally, phenolic compounds increase functional value of the bread but may have negative effects on taste, texture, and appearance concentration, so that optimization is necessary to balance health and consumers’ acceptability [49,55].

In bread production with the addition of broccoli stalk powder, ΔE serves as a useful parameter for evaluating color changes relative to the control bread. All samples containing BSP exhibited high ΔE values, indicating pronounced chromatic differences. Broccoli stalk powder (BSP) contains pigments such as chlorophyll and phenolic compounds, which can contribute to greener or darker color tones, reduced lightness (darker crumb), and additional color changes during baking, including those associated with Maillard reactions. While ΔE objectively quantifies these differences, the bread does not merely appear “greener.” For bread formulated with functional ingredients, higher levels of supplementation may be acceptable, provided the product is appropriately positioned and evaluated within the CIELAB color space.

According to Nikolov et al. [63], two thresholds can be mentioned separately: if ΔE < 1, the color difference is imperceptible, while if ΔE > 3, it is visible. In the present work, all ΔE values are greater than 5, so the color difference is clearly visible and significant compared to the control bread. There was no linear increase between the percentage of BSP addition and ΔE. Bread with 5% BSP had a lower ΔE than bread with 3% and 7% BSP, respectively, possibly as an effect of the non-uniform distribution of pigments and/or chemical reactions (Maillard/oxidation). A recent study clearly supports the threshold ΔE > 3 as a perceptible difference in bakery products [44]. In the same study, ΔE ranged between 5.32 and 15.18 for reformulated breads containing broccoli extracts. Previous studies reported an increased green hue in broccoli-enriched crackers. The final product showed a ΔE value greater than 3, indicating a perceptible color difference compared with the control [31].

The incorporation of BSP resulted in high ΔE values, which were also accompanied by a reduction in the bread loaf volume. Although this relationship is not mathematically direct, it suggests a concurrent influence of BSP on both the crumb color and bread structure. The addition of 3% BSP resulted in a relatively high ΔE value (10.89), while the overall sensory score was comparable to that of the control sample, indicating no clear improvement in organoleptic quality. The incorporation of 7% BSP into wheat flour produced the highest ΔE value (11.61) but also the lowest sensory score among the tested formulations. The bread color was perceived as excessively intense (greenish/dark), which may have been associated with a perceived product defect. In contrast, the incorporation of 5% BSP resulted in a comparatively lower ΔE value (9.27) and the highest sensory score (3.34). The modified color of the bread was acceptable or even pleasant, possibly perceived as “natural/healthy”, which suggests that this addition proportion, 5% BSP, is optimal. Spearman’s rank correlation confirmed a perfect monotonic relationship (ρ = −1.00, *p* < 0.01), indicating that higher color differences consistently correspond to lower sensory acceptance.

Overall, the results suggest that BSP addition significantly influences both the physical and sensory properties of bread, highlighting a trade-off between nutritional enhancement and product acceptability. The 5% BSP level can be considered optimal, as it provides a balance among color changes, loaf volume, and sensory acceptance.

## 4. Materials and Methods

### 4.1. Material

Broccoli (*Brassica oleracea* L.) was purchased from a local market in Romania. The florets were removed, and the stalks were washed and cut into cubes of approximately 1 cm on each side. The prepared vegetable material was freeze-dried (Biobase BK-FD10P, Biobase Bioindustry, Jinan, China) and subsequently powdered using a laboratory mill. Care was taken during powdering to avoid heating the material.

### 4.2. Dough and Bread Making

All raw materials used in the bread formulation, including wheat flour, yeast, and salt, were purchased locally.

The flour used in the formulation of both control and experimental breads was wheat bread flour type 00 (average moisture content 13.56% wb, 0.6% ash content, 12% protein). Broccoli stalk powder (BSP) was incorporated into the wheat flour at levels of 3, 5, and 7%, as shown in Table 7.

For each experimental batch, the dough was mixed for 7 min in a spiral mixer (MB-1300, Biovita, Cluj-Napoca, Romania). Mixing was performed in two stages: homogenization and actual mixing. The bread was produced using the direct method.

The dough batches were fermented in trays at 30–32 °C for 40 min, with a relative humidity of air by 75%.

The dough was baked in molds in an electric oven (Zilan, model ZLN2433, Istanbul, Turkey) at 180 °C for 25 min. After baking, the breads were removed from the molds and cooled rapidly.

### 4.3. Physico-Chemical Analysis

All chemicals used were purchased from Merck (Darmstadt, Germany).

Flour and bread moisture contents were determined by measuring the weight loss of the sample after drying at 130 ± 1 °C for one hour, until a constant mass was achieved.

The water-holding capacity of the wheat flour and BSP was determined by hydrating 1 g of sample for 30 min, followed by centrifugation for 5 min at 1000 rpm (EBA 21 Hettich Zentrifugen 1004, Tuttlingen, Germany).

The wet gluten content of the wheat flour was determined by washing the dough prepared from the flour sample with sodium chloride and drying off the obtained gluten. For the calculation, the quantity of retained water is divided by the amount of the sample.

The acidity of bread, defined as the total of acids and acid-reactive compounds present, was determined via titration with a base.

Fornet equipment (device reproducing Bread Volume Meter 13300, in-house developed system, Valahia University of Targoviste, Targoviste, Romania) was used for the determination of the loaf volume, as described in the standard AACC method 10-05.01 [64].

Porosity represents the total volume of pores within a given volume of bread crumb, expressed as a percentage. The total pore volume was determined from a known crumb volume, based on its mass and density. Portions of the crumb were cut from different points of the crumb section using a cylindrical perforator, and their volumes were calculated to obtain an average sample, as porosity of the bread varies across the section.

Porosity, together with pore structure (uniformity and size) and wall thickness, is an important indicator of product quality, which largely affects the digestibility of the bread.

Elasticity values were determined by pressing a piece of bread crumb of a specified shape for a set period and measuring its return to the original position after the force was removed. The elasticity of the bread core is expressed as a percentage, calculated as the ratio between the height after pressing and recovery and the initial height of the crumb cylinder.

The Brabender Farinograph (Brabender GmbH & Co., KG, Duisburg, Germany) was used to measure the rheological properties of the dough in the time of the mixing, as described in the standard AACC method 54-21.02 [65]. The Chopin Alveograph (Alveolab, KPM Analytics, Westborough, MA, USA) was used to measure the quality of flour and the behavior of dough during fermentation by simulating the leavening process, as described in the standard AACC method 54-30.02 [66].

The Falling Number (FN) was determined by measuring the time (in seconds) needed for a piston to fall through a gel formed from a suspension of flour and heated water, according to the AACC method 56-81.03 [67].

Total dietary fiber in broccoli stalk powder and enriched breads was determined according to the AOAC 985.29 enzymatic gravimetric method, using the Total Dietary Fiber Assay kit from Megazyme (K-TDFR-100A/K-TDFR-200A 04/17, Megazyme International Ireland Ltd., Bray, Co., Wicklow, Ireland). The method involves enzymatic digestion followed by gravimetric determination of the residue.

Hydroalcoholic extracts of the samples were obtained via ultrasonication. Briefly, a solvent of 50% ethanol (absolute ethanol:double distilled water, 1:1, *v*/*v*) acidified with 0.1% (*v*/*v*) concentrated hydrochloric acid, was added to the material at a ratio of 0.5:10 (material: solvent). Extraction was performed via ultrasonication (PRO 70, 40 kHz, ASonic, Shenzhen, China) at room temperature for 20 min, with intermittent agitation. The supernatant was separated via centrifugation during two stages of extraction.

The total phenolic content (TPC) was determined using the Folin–Ciocalteu method. To 0.5 mL of extract, 10% Folin–Ciocalteu reagent was added with intermittent stirring, followed by 2 mL of 8% Na_2_CO_3_ solution. After 60 min at room temperature, the absorbance of the samples was measured against a blank sample at 765 nm (UV-VIS spectrophotometer model UV 1720, Shanghai Yoke Instrument Co., Ltd., Shanghai, China). The results were expressed as gallic acid equivalents (GAE).

The antioxidant activity of BSP and bread was determined using the DPPH method. To 0.75 mL of extract, 1.5 mL of DPPH solution was added and vortexed. The samples, along with a DPPH blank (A_blank DPPH_) and an extract blank (A_blank extract_), were kept at room temperature in the dark for 30 min. The absorbance of the samples (A_sample_) was measured against ethanol as a control at 517 nm. The results were expressed as follows:

$$\mathrm{AA}\%\;=\;\frac{\left[A_{blank\;DPPH}-\left(A_{sample}-A_{blank\;extract}\right)\right]}{A_{blank\;DPPH}}\times 100$$

The color of the samples was measured in the CIELAB system using an NR 110 Precision Colorimeter (3nh-ThreenhTechnology Co., Shenzhen, China), equipped with CQCS3 software version 3.4.3, a D65 light source, and a 4 mm aperture. The following color parameters were recorded: lightness (L*, 0 = black, 100 = white), redness (a*, <0 = green, >0 = red), and yellowness (b*, <0 = blue, >0 = yellow).

The overall color difference (ΔE) for each formulation with BSP supplementation relative to the control bread was determined using the following equation:

ΔE = (Ls−Lc)^2+(as−ac)^2+(bs−bc)^2

where the index “s” refers to the color components of the sample with BSP addition and the index “c” to the control sample.

### 4.4. Small-Scale Consumer Test

A small-scale consumer test was conducted on the bread samples. The panel consisted of 23 untrained assessors (aged 20–35 years), who evaluated overall acceptability using a five-point hedonic scale, ranging from 0 (dislike extremely) to 4 (like extremely).

### 4.5. Statistical Analysis

Experimental data are presented as means ± standard deviations (*n* = 3). Statistical analysis was performed using IBM SPSS Statistics 26. One-way Analysis of Variance (ANOVA) was conducted to evaluate the effect of the BSP addition level on the measured parameters (*p* < 0.05).

Pearson correlation analysis and Principal Component Analysis (PCA) were performed using the correlation matrix to gain deeper insights into the quality of the breads.

## 5. Conclusions

The incorporation of by-products into new food products allows for reduced production costs, lower waste-management expenditures, and the creation of new economic value streams. In line with the United Nations Sustainable Development Goals, converting by-products into functional ingredients contributes to the development of sustainable consumption and production patterns [7].

Broccoli stalk is a valuable by-product of the food industry, so its use can contribute to reducing food waste, valorizing unused nutritional components, and the development of functional products.

The moisture, acidity, porosity, and loaf volume of bread were significantly affected by the addition of broccoli stalk powder. The dietary fiber in broccoli stalk not only increases the total fiber content of the bread but also influences its technological, sensory, and nutritional properties. The incorporation of 5% freeze-dried broccoli stalk into wheat flour with a balanced rheological profile and gluten system resulted in a 50% increase in the total polyphenol content and an 8% increase in antioxidant activity compared to the control bread. Sensory acceptability decreased only at the highest level of broccoli stalk powder supplementation (namely, 7%) in the recipe.

Enrichment of the wheat flour with 5% broccoli stalk flour was identified, based on the evaluated parameters, as providing the optimal balance among functional enhancement, sensory acceptance, and the bioactive quality of bread. Further research is needed to investigate the rheological behavior of the dough, shelf-life stability of the bread, and the bioactive compounds present in the product. A complete characterization will support the recommendation of broccoli-stalk-flour-enriched bread for industrial applications. Special attention should be given to the dynamics of bread parameters during storage, as well as to the in vitro simulated digestion of the bioactive compounds.

## Figures and Tables

**Table 1:** Characterization of wheat flour and broccoli stalk powder.

Sample	Moisture (%)	Hydration Capacity (%)	Wet Gluten (%)	DI (mm)	I_e_ (mm)	Acidity (°A)
Wheat flour	13.56 ± 0.05	58.66 ± 0.57	24.86 ± 0.11	4.06 ± 0.11	21 ± 0.09	2.56 ± 0.05
Broccoli powder	6.23 ± 0.01	290 ± 2.8	NA	NA	NA	5.6 ± 0.2

NA—not applicable.

**Table 2:** Correlation of farinographic and alveographic parameters.

Farinographic Parameter	Value	Observations	Alveographic Parameter	Observations
Water absorption	56	Moderate hydration, easy-to-work dough	W = 188 × 10^−4^ J (average energy)	Moderate water retention capacity and gluten development
Dough developing time	2.8 min.	Rapid gluten development	L = 80 mm, P/L = 0.85	The gluten network forms quickly, corresponding to moderate extensibility
Dough stability	5.4 min.	Medium stability; low tolerance to kneading	P = 68 mm H_2_O	The dough has moderate strength, according to medium stability; does not tolerate long mechanical stress
Dough softening	92 FU	High softening; dough loosens quickly	G = 19.9	The gluten network begins to relax and loses elasticity rapidly; correlated with the high degree of softening
Quality number	55	Average quality, suitable for standard baking	W = 188 ×10^−4^ J, P/L = 0.85	Energy and P/L balance confirm the moderate performance of the flour

W—baking strength; L—extensibility; P—tenacity (resistance to deformation); P/L—ratio between dough strength and extensibility; G—inflation index.

**Table 3:** Physico-chemical parameters of experimental bread.

Sample	Moisture (%)	Acidity (°A)	Porosity (%)	Loaf Volume (cm^−3^)	Elasticity (%)
BC	41.99 ± 0.02 ^a^	2.7 ± 0.01 ^a^	92.1 ± 0.05 ^a^	320.66 ± 1.15 ^a^	95.31 ± 0.02 ^a^
B3BSP	44.39 ± 0.08 ^b^	2.66 ± 0.05 ^a^	72.46 ± 0.46 ^b^	284 ± 4.0 ^b^	92.78 ± 0.17 ^b^
B5BSP	45.86 ± 0.14 ^c^	3.1 ± 0.01 ^b^	71.16 ± 0.11 ^b^	265 ± 3.0 ^c^	96.63 ± 0.23 ^c^
B7BSP	47.28 ± 0.33 ^d^	3.33 ± 0.05 ^c^	72.69 ± 0.11 ^c^	238.66 ± 1.52 ^d^	97.44 ± 0.2 ^d^

BC—control bread; B3BSP—bread supplemented with 3% (*w*/*w*) broccoli stalk powder; B5BSP—bread supplemented with 5% (*w*/*w*) broccoli stalk powder; B7BSP—bread supplemented with 7% (*w*/*w*) broccoli stalk powder; different lowercase letters within the column and for each group of samples represent significant differences according to Tukey’s-b test (*p* < 0.05).

**Table 4:** Colorimetric parameters of bread crumb samples.

Sample	L*	a*	b*
BC	52.39 ± 0.12 ^a^	5.83 ± 0.14 ^a^	11.15 ± 0.06 ^a^
B3BSP	60.75 ± 0.12 ^b^	−0.94 ± 0.08 ^b^	12.89 ± 0.09 ^b^
B5BSP	56.6 ± 0.3 ^c^	−1.89 ± 0.01 ^c^	14.10 ± 0.12 ^c^
B7BSP	55.1 ± 0.09 ^d^	−2.99 ± 0.1 ^d^	18.2 ± 0.09 ^d^

BC—control bread; B3BSP—bread supplemented with 3% (*w*/*w*) broccoli stalk powder; B5BSP—bread supplemented with 5% (*w*/*w*) broccoli stalk powder; B7BSP—bread supplemented with 7% (*w*/*w*) broccoli stalk powder; L*—lightness; a*—greenness; b*—yellowness; different lowercase letters within the column and for each group of samples represent significant differences according to Tukey’s-b test (*p* < 0.05).

**Table 5:** Pearson correlation analysis of key parameters.

	Moisture	Porosity	Elasticity	Volume	Acidity	AA	TPC	Lightness	TDF
Moisture	1	−0.844 **	0.547	−0.993 **	0.878 **	0.893 **	0.795 **	0.275	0.947 **
Porosity		1	−0.089	0.830 **	−0.515	−0.808 **	−0.645 *	−0.719 **	−0.803 **
Elasticity			1	−0.548	0.866 **	0.247	0.728 **	−0.617 *	0.552
Volume				1	−0.870 **	−0.913 **	−0.764 **	−0.269	−0.940 **
Acidity					1	0.663 *	0.825 **	−0.205	0.864 **
AA						1	0.459	0.459	0.834 **
TPC							1	0.013	0.782 **
Lightness								1	0.256
TDF									1

** correlation is significant at the 0.01 level; * correlation is significant at the 0.05 level.

**Table 6:** Component Score Coefficient Matrix.

Rotated Component Matrix ^a^		
	Component	
	1	2
Moisture	0.982	0.156
Porosity	−0.921	0.332
Elasticity	0.418	0.905
Volume	−0.977	−0.153
Acidity	0.791	0.600
Antioxidant activity	0.908	−0.140
TPC	0.765	0.438
Lightness	0.433	−0.888
TDF	0.951	0.176

Extraction method: Principal Component Analysis; rotation method: Varimax with Kaiser Normalization ^a^ rotation converged in three iterations.

**Table 7:** Experimental variants of bread.

Sample	Wheat Flour (g)	Water (mL)	Yeast (g)	Salt (g)	Broccoli Stalk Powder (g)
Control	500	320	20	7.5	-
Bread with 3% broccoli (B3BSP)	500	320	20	7.5	15
Bread with 5% broccoli (B5BSP)	500	320	20	7.5	25
Bread with 7% broccoli (B7BSP)	500	320	20	7.5	30

## Data Availability

The original contributions presented in this study are included in the article. Further inquiries can be directed to the corresponding author.
